# Lectures on the Theory and Practice of Physic

**Published:** 1840-01

**Authors:** 


### REVIEWS.

Art. IV.—*Lectures on the Theory and Practice of Physic, delivered in the College of Physicians and Surgeons of the University of the State of New York. By the late* David Hosack, M. D. L. L. D., F. R. S., *Professor of the Theory and Practice, and of Clinical Medicine in that Institution. With an Introductory letter, by* Nathaniel Chapman, M. D., *Professor of the Theory and Practice of Medicine, in the University of Pennsylvania, &c. Edited by his friend and former pupil,* Henry W. Duchachet, D. D., *Rector of St. Stephen's Church,* Philadelphia. Herman Hooker, Chesnut street, 1838. p. p. 699, 8vo.

The author of this volume was classed, during his life-time, with the ablest teachers of medicine in the United States; and its recommender and endorser holds, at present, a similar rank. We are justified, moreover, in alleging that the production of the volume is the result of the labor of not less than *thirty years*, industriously employed by the writer in observation, reading, composition and thought. It is therefore a work of high authority, and must be regarded as presenting a full and fair view of the treatment of diseases in the Atlantic States, as well as of the style and manner *in* which, or at least of the doctrines and directions *through* which, their treatment is taught in the medical schools of that portion of the United States.

Did any doubt exist as to the correctness of these remarks the commendations contained in Professor Chapman's "Introductory Letter" would dispel it. In his reference to the "Lectures" of which the volume consists, the Professor says:

"They embody a very large mass of curious and *useful* information, *clearly* and *agreeably conveyed.* Excepting some *pathological doctrines* I find little in them to which I would object. *The practical part I consider sound, or at least it corresponds very much with my own views.* Long and extensively engaged in the profession, and with his acute and discriminating mind, he, (Professor Hosack) could scarcely fail to arrive at just conclusions in whatever regards the management of disease. He deserves, and will no doubt rank, among the most authoritative of our writers. You (the Editor) have done well in publishing the work. *No effort shall I spare to promote its distribution; and I mean especially to recommend it to the attention of my class.*"

Such are the high and imposing auspices and recommendations to favor, under which this volume is ushered to the world. Is it worthy of them? and does it fill up completely the measure of expectation and confidence they are calculated to excite? Without assuming the responsibility or claiming the prerogative of giving to these questions categorical answers, we shall endeavor, by a brief discussion of the matter they embrace, to enable our readers to answer them for themselves.

Like all other productions of the kind, this which lies before us consists of an exposition of principles, and a system of practice. And with neither the one nor the other do *we* find reason to be satisfied; at least in their relation to this section of country where we ourselves reside, and respecting which alone we presume to decide. Whatever may be their amount of *aptitude* and *value* in the Eastern States, (and Pro fessor Chapman is earnest in their praise) we are greatly mistaken if they have much of *either* in the States of the West. We do not positively say that they are better fitted to do mischief than good in this part of the Union; but we do say that nothing could tempt us to teach or recommend them to western students of medicine; and we should deeply grieve to see a sick friend treated in conformity to them. In truth they would be, of themselves, sufficient to convince us, were we not already convinced by other considerations, which have long been familar to us, that the Professors of the Atlantic schools are but very moderately qualified, or rather not qualified at all, to communicate to their pupils such views of *practical* medicine, as are suited to the diseases of the Mississippi Valley. How indeed can the case be otherwise? How is it possible for those professors, however able and learned they may be, to teach successful modes of treating diseases which they have never seen, and of whose ruling and peculiar features and character they are necessarily uninformed? In fact they neither do possess nor can possess, such resources for teaching. Nor can they ever attain them, unless they pass the mountains, and spend years in the midst of us, observing, studying, and treating our complaints. Let them thus act, and thus accomplish themselves, or else confine their instructions to the cure of maladies with which they have been conversant, and detail them only to the medical pupils of their own region For *practical* purposes in the West, those instructions have neither fitness nor value. However *sectional* and exceptionable these sentiments may be considered in some places, and represented by some Journalists, they are too true and plain to be candidly *opposed by either fact or argument*, though they may be sturdily *denied*, and dogmatically *condemned*. And sufficient evidence to this effect shall be presently adduced. But a few farther introductory remarks must be previously offered.

In matter as well as in manner, the book we are examining is in accordance only with by-gone days—with some period we mean of the *eighteenth century.* Were its title "A Tale of other Times," it would not be inappropriate. Had it been published in 1788 (*fifty* years ago) instead of 1838, and in Germany or Prussia, instead of the United States, it would have been somewhat in harmony with the period and the place, and with the then-existing condition of the medical p1ofession. But issuing from the American press at the *present* period, with the substance which makes it up, the shape which marks it, and the parade and ostentation put forth in its preparation, it is one of the most ill-timed, inappropriate, and *dislocated* productions we have ever witnessed. It is in manner, morever, a thing entirely apart from the present age—too starched and stiff, formal, dogmatic, and authoritative, for the free and easy, and flexible spirit of 1839. It is as much out of fashion, and as ill-suited to the medical taste and habit of the present day, as would be the bag-wig and the three-cornered hat for the medical head, or the flowing, plaited, and tasselled gown for the Professor's shoulders, when he presents himself to his class. It is much more characterized too by research and learning, than by judgment, science, and experience—especially that judgment and that experience, which could be made available in the treatment of our *western* diseases. Nor is this all. Though we shall not pronounce it a work of pedantic ostentation and learned parade; yet, were we to do so, to convict us of error would be a difficult task. We venture, however, to say, that a student of medicine may pore over such a book, or listen to the reading of it in the form of lectures, until he has memorized every word of it, and still be no better qualified to become a *successful practitioner* in the Mississippi Valley, than he would be by simply reading over

Boerhaave's aphorisms, Cullen's Outlines, or Gregory's Conspectus. And neither of these measures, nor any degree of attention he can pay to *eastern* lectures, or to the reading of European books, published during the last century, nor all of them combined, can fit him so well for the cure of western complaints, as will a faithful attendance on even a *single course* of *practical* lectures, prepared with ability and judgment, and well delivered, by a *western* teacher, who is thoroughly versed in the knowledge and treatment of such diseases.

While they manifest a state of marasmus, as respects originality, and a profound investigation of the laws of living organic matter, as they show themselves in the human body, in the phenomena and functions of health and disease, these "Lectures" are *hypertrophied* or *dropsical* in references, authority, and learned quotation. And this is true as respects almost every topic of which they treat. Whatever of thought they may contain is so enveloped in studied erudition, as greatly to obscure it, and so interwoven with the numerous opinions of other writers, as to puzzle the reader, and sometimes defeat him in his efforts to disentangle it. Much more deeply and annoyingly must it have puzzled a class of pupils, but slightly versed in medicine, to follow, comprehend, remember, and digest such a mass of learned matter as the "Lectures" contain, when they were merely read to them. The Professor seems never to have forgotten either himself, his authorites, or quotations, in his devotion to his subject. Nor is the matter at all mended by the fact, that such authorities are mostly the out-of-print and inaccessible works of ancient and early-modern writers, and the quotations too frequently in the Latin tongue. To *recent modern* authorities (those I mean that have appeared within the last twenty or thirty years) his references are comparatively few. In proof of these remarks, a few special notices of the "Lectures" shall now be given. We shall begin with the Professor's Nosology, in which he has labored technically and abstractedly, and threaded a tangled wilderness of nomenclature, to a great extent, but, in our opinion, as regards utility, to very little purpose. Though we shall not exclaim, with the late Professor Rush, "delenda est nosologia" —let nosology be expunged from medicine; yet, while it is certainly the most difficult and repulsive branch of the profession, we regard it as by far the least instructive and useful one.

This subject, we say, our author has labored to an extravagant and unprofitable extent. His definition of it moreover we consider erroneous and deceptive. He denominates it an "arrangement of diseases in such order as will be best calculated to *give us a knowledge of them.*" To this "definition" (if so it can be called) we confidently reply, that a mere "*arrangement*" of diseases gives us *no* "knowledge of them." It gives us uninstructive *names* and *scholastic places* for them, and nothing more. When we have acquired, through other channels, a "knowledge of diseases," these names and places serve the purpose of remembrancers; and there their usefulness ends. As respects all other things the same is true. The things themselves, whatever they may be, must be learnt *first*, else their names are but empty and unprofitable sounds. True, in the nomenclature of diseases now forming, there is somewhat more of meaning and common-sense. The reason is plain. An effort is making to render the "sound an echo to the sense"—to affix a name which will express the leading characteristic of the complaint it designates. Yet still is the instruction thus conveyed exceedingly limited.

Names however must be given; but they should never be relied on for "knowledge."

As is his uniform custom, in relation to every topic he touches, our author crowds his lecture on nosology with all the names of nosologists he is able to muster. Nor does he observe in the citation of them any regular chronological order. He begins we think with Sydenham and Baglivi. Then come Aretæus, and Cœlius Aurelianus; Johnston, Sennertus, and Morgagni; Mead, Good, and Chapman; Boerhaave, Riverius, Hoffman, and Sauvages; Linnæus, Vogel, Sagar, McBride, and Cullen; with some others perhaps of minor note.

Cullen's Synopsis nosologiæ the Professor details at full length, consisting of four classes, eighteen orders, or perhaps more, and one hundred and fifty genera. To many points of this synopsis he offers objections, and then favors us with his own nosology. This contains eight classes, twenty-four orders, and one hundred and ninety-eight genera. Professor Chapman, as we have already shown, assures us, in his "Introductory," and recommendatory "letter," that, certain pathological doctrines excepted, he approves of the volume before us, by the lump. But nosology has no connexion with pathology. Of course the able Philadelphia Professor sanctions the nosological arrangement of Professor Hosack, with its two hundred and thirty significant appellatives! And we ask him seriously and respectfully, whether the fearful task of the commitment to memory of this long catalogue of "hard words," supposing it to be mastered, would furnish a pupil with a tittle of knowledge that would be useful to him, either in the true *science* of medicine, or in the management of disease? Nor will he venture to answer our question in the affirmative. And we ourselves very confidently affirm, (without the least apprehension of being contradicted, and without regarding contradiction, should it occur) that a load on the memory, so multifarious and weighty, as Professor Hosack's nomenclature must produce, would give no shadow of aid in the cure of *western* diseases. Far from it. On the contrary, a crop of weeds so dense and useless, perhaps we might say so decidedly noxious, would be much more likely to choke and stint some salutary plants, or expel them entirely from the garden of the mind.

The next portion of the volume we are considering, on which we shall offer a few remarks, is the lecture "on fevers in general." This lecture we fix on for our purpose, not by way of selection, but because it comes next in the course of the book.

Here we have another display of learning to excite our wonder, and another amasment of authority to edify us. After referring to Hesiod, and quoting Horace, and speaking most learnedly of Pandora and her box, our author turns lexicographer, and favors us with a knowledge of the derivations and classical meanings of *fever*, *symptom*, *pathognomonic*, *diagnosis*, and perhaps a few other terms of Greek and Latin origin. His next attempt is to *define* fever. And here he summons from the grave, and from all times and places, a cloud of witnesses so dense, self-conflicting, and impenetrable, that instead of aiding him in the exposition and illustration of his subject, they do little else than obscure and distract his own views, and his reader's conception, by diffusing discord and darkness around him.

After duly descanting on the doctrines of fever maintained by Cullen, Boerhaave, Sauvages, and other moderns, and dissenting from each and all of them, he goes back to a remoter period of medical history, and musters, as auxiliaries in the display he is making, the opinions of ancient physicians, and of those of an early date in modern times. In these references, that he may really "begin at the beginning," or as near to it as possible, he introduces us, as a matter of course, to Hippocrates, but declines making us known to Æsculapius or his sons. He then passes in review before us, like the descendants of Banquo before the eyes of Macbeth, the shadowy forms of Erasistratus, Herophilus, Aretæus, and Celsus; Galen, Sennertus, Vogel, Van Helmont, De Haen, Pringle, Lind, Fordyce, Clutterbuck, and others.

We said that our author invoked all these worthies to aid him in discovering the nature of fever, and framing for it a definition. In this statement however we were mistaken. The Professor summoned those writers before him, to charge them with error, and show them that their views of fever were incorrect, and that he himself was the oracle to instruct them. And the precious revelation he makes to them is as follows:

"Fever I define to be, an affection of the whole system"! (Page 57.) To convince them that this revelation is genuine, and to induce them and all the living world to believe it emanated from himself, and was new to everbody else, he reveals a further fact, in the following words: "*My* definition, that fever is a disturbance of all the functions is adopted by Dr. Wright, Professor Jackson of Boston, and by Dr. Potter of Baltimore; and indeed the same is now adopted substantially in the Philadelphia school."!

Such is Professor Hosack's assumption of originality, as relates to the essence or definition of fever. And we regret that he made it; because it is groundless. That definition, whether right or wrong—a true revelation or a false one—belonged no more to him, than it does to ourselves. Professor Rush announced it *forty years* ago. Nor are we willing to vouch that it was original with him. We suspect that it was not. He often however in his lectures thus expressed himself "Gentlemen, fever is a *disease of the whole system.*" And in passing from one form of fever to another, he frequently, if not uniformly remarked:—"This also, gentlemen, is a disease of the whole system."

But no matter who is the author of this so called definition of fever; and no matter who adopts it. Two objections lie against it, either of which is fatal to it. It is not a *definition* of any form of fever, because it communicates no *definite idea* of any sort of such affection—no idea, we mean, by which the complaint can be known and distinguished, by those who have received no knowledge of it from any other source. And an objection still more formidable is, that the definition *is not true.* There are forms and degrees of genuine fever, in which *all* the functions of the system are not deranged. This is true as respects the *external senses.* They are very important functions; and yet in various forms and degrees of fever they are as sound as they are in health. Indeed the cases of fever, in which *all* the external senses are "disturbed" *from the beginning*, are but few. In an *advanced stage* of very severe cases of cephalic fever, all the external senses often suffer; but rarely from the *commencement* of them. Were we inclined to dwell on this subject, we might say the same of the intellectual and moral faculties, whose soundness depends on the soundness of the brain. In many cases of fever they also are not disturbed—neither strengthened, debilitated nor perverted.

Our author then, we repeat, in his attempt to define fever has not been successful. Nor has any other writer. Fever has never been defined, notwithstanding the numerous efforts that have been made to that effect, by the ablest and most eminent members of the profession. Worse still; it never can be defined; because, in different stages, different types, and different cases, it is widely different from itself—and *still is fever.* A *correct* definition of it, therefore, in one form, or one stage, would be an *incorrect* one in another. Hence, we say, a full and satisfactory definition of fever, in the abstract, can never be framed. The only practicable way to give a true picture of it is to delineate its several stages or elements in succession, and unite them as a whole—and the work will then be done—as well at least as it *can* be done.

Should any of the friends of our author, or any of those who concur with him in opinion, and are anxious to promote the circulation of his "Lectures," take exception at the freedom of our strictures on them, and the unceremoniousness of our objections to their doctrines, our reply to them is brief. We believe our objections and strictures to be true, and that it is our duty to offer them. Nor is this all. Professor Hosack in person, as his "Lectures" testify, has set us the example. He was himself proverbially a *fault-finder.* He rarely referred to a *contemporary* writer or teacher, but to state objections to some of his doctrines. It was chiefly on the opinions of the distinguished dead alone, especially of those who died centuries ago, that he did not make war, but quoted them as authority.

Lecture VI. on "Fevers in General," is a singular production. Though it contains a large amount of matter, not in itself uninteresting or useless, yet that matter is thrown together in a manner so irregular, loose and disjointed, as to present to the reader no definite end or aim, much less to establish any given point. In this Lecture moreover the Professor strikingly manifests and establishes the fact, that he is, in a high degree, a writer of other times, by his continuing to be a believer in antiquated and *obsolete notions.* Of this adherence by him to cast-off errors, either from obstinacy, or a want of better information, the following is an instance, the existence of which we would not have credited, had we not found it in his book.

"As before observed, it is the effect of respiration to increase the heat of the body—*the lungs are the fire-place*"!—In 1788, not long after the discovery of *latent heat,* this notion might have been tolerated; because it would have been in accordance with the philosophy of the day. But in 1838 (the date of the Lecture) it is equally in opposition to existing philosophy and established truth, and therefore unpardonable. Respiration does *not* increase the heat of the body; and the lungs are no more the fire-place, than the stomach or the liver. The notion bespeaks a radical defect in the Professor's physiology.

But from matters merely doctrinal, we shall pass to those that are strictly practical. And we regret to observe, that here also our report of the Lectures will not be commendatory—especially as relates to the treatment of diseases in the Valley of the West. We shall select for our purpose, the practice recommended by Professor Hosack in such forms of disease as are most prevalent in that Valley. Fever, in some of its modifications, is a disease of this description. That the Professor's mode of treating this complaint therefore may be seen, examined, and understood as it is, without abridgement, change or misrepresentation, we shall lay before the reader his nineteenth "Lecture" entire, whose heading is,

"THE GENERAL TREATMENT OF FEVER—TREATMENT OF THE FIRST STAGE."

"The treatment of fever now falls under our consideration; and, as in describing the phenomena of fever we divided it into its different stages, we shall observe the same order in speaking of its treatment, pointing out, as far as may be practicable, the remedies proper to be employed in the different stages of fever. At this time I propose, therefore, to point out the general indications of cure, the general means of fulfilling those indications, and the principles upon which those means are to be employed.

"In the treatment of the first stage, constituting the invasion of fever, the first indication, as we have remarked, agreeably to our view of the proximate cause, is to counteract the irritation, which appears more especially in the nervous system. Dr. Cullen's indication, agreeably to his view of the proximate cause of fever, ought to be to counteract his supposed debility, and accordingly, bark and wine, with other tonics and stimuli, would be the best remedies; but neither his doctrine, nor the treatment it leads to, I trust, will be contended for. The irritation we have noticed as existing in the first stage, or rather as constituting the first stage, shows itself in various ways. The symptoms which usher in the first stage of fever may be divided into three classes.

"The first class we may denominate the ordinary attack of fever, exhibiting itself perhaps by a slight chill and some sense of coldness, but without much pain or any other symptoms, local or general.

"In the second class, we find the patient attacked with severe pain, showing itself in the head, back, or limbs, or in all at the same time, with great general soreness and stricture of the surface—a distressing sense of coldness amounting to rigors or perhaps convulsions. These rigors are sometimes fatal, especially to aged persons; but Dr. Cullen remarks, that rigors taking place, the patient is not carried off by that paroxysm. This is not always so. I recollect the case of a gentleman who was thus attacked with rigors upon the invasion of a paroxysm followed by stupor, and which proved fatal to him during the very paroxysm—the irritation was such that they ended in an apoplexy; such was the crowded state of the vessels of the brain. He was subject to intermittents, and they usually affected his head. He had been exposed to the cause of it; it occurred during the season of its prevalence, and from the rigors and general symptoms which ushered it in, the character of the disease was not to be doubted. This event too, I believe, is of much more frequent occurrence than is usually supposed; it might be called the apoplectic state of intermittents.

"The third class of symptoms we find still more formidable and alarming, viz. delirium or mania; for all fevers, as we have seen is the case with yellow fever and the plague, are not ushered in by the ordinary chill that usually announces the paroxysm of an intermittent.

"In the ordinary attack of fever, the heat generating power of the system is sufficient to counteract the temporary effects of the irritation produced upon the exteme vessels, for the tone of the system is not impaired, nor are any of the vital organs particularly oppressed. In that case, the constitution being good, and the body not more than ordinarily cold, the common practice is generally sufficient, viz. to place the patient comfortably warm in bed, and to administer to him some warm tepid drinks, as various teas or toast water.* Weak mint or catmint tea, when they can easily be procured, are preferable drinks when the stomach is disturbed during the invasion of fever. They should, however, be taken of moderate strength, as they may otherwise, by the essential oil they contain, increase the excitement of the system; the quantity too should be regulated by moderation, for we may excite the system by the quantity as well as the quality of the drinks we employ. I frequently also direct the patient previous to his getting into bed, if his extremities be cool, to bathe the feet in tepid water. I then give him a moderate sudorific anodyne, composed of ℥ss. of the spiritus mindereri, with from fifteen to twenty-five drops of laudanum. If the stomach is irritable, administer the dose in mint water.

"It is important, however, to remember that the hot stage of fever is very soon to succeed. Keep this always in view in all your prescriptions. On this account, too, observe the temperature of the water in which the patient immerses his feet, of the drinks he employs, and the temperature of the air of the room. With the same view the quantity of his bed clothing should also be regulated by the physician, and all stimuli, such as light, noise, company, business, should be withdrawn or guarded against at this time, in order to prevent or moderate the excitement which is soon to succeed.

"But one of the most important and efficacious class of remedies which can be administered at the invasion of fever, (especially the remittent and continued forms of fever,) when the situation of the patient is such as to call for or to justify

*Let me here remark, once for all, that the proper mode of makiug toast or bread water, is by infusing the slice of bread, when well browned, in boiling water, but not in cold water, as it usually is done; for the object is to give the drink moderately warm, and to render it grateful to the nauseated stomach; to do so, this is the only proper mode of preparing this drink, and thus prepared, it is one of the most palatable and grateful drinks to patients in general that can be directed in an irritable stomach, at the same time that it is readily obtained.

their use, is *emetics*. Armstrong, a late writer, p. 99, observes that 'in the beginning of almost all febrile complaints, emetics will generally be found very beneficial, though much neglected now-adays by many practitioners.' The emetic having operated, I usually direct some Indian gruel, either sweetened with brown sugar, or seasoned with a small quantity of common salt, to be taken afterwards, with the view to obtain some effect from it upon the bowels; in some instances, an hour or two after the emetic has ceased to operate, I add the sulphate of magnesia, or the sulphate of soda, or Rochelle salts to the gruel, or solicit an evacuation from the bowels by an enema, or a dose of Seidlitz powder.

"In the first stage, where it occurs under ordinary circumstances without those peculiar distressing impressions upon the nervous system; without the inordinate fulness of the blood-vessels producing delirium, stupor, or mania; where the degree of cold is not excessive; where the patient is neither too plethoric nor of too feeble a habit, nor too advanced in life, when the circulation of the brain is less active, and the vessels of the brain are perhaps loaded by the venous plethora; when these circumstances forbid their use, emetics, and these given so as to produce full vomiting, may be directed with the best effects. In some instances, too, the breath of the patient is offensive, the tongue is foul and loaded with sordes; he complains of a disagreeable taste in his mouth; in such cases, whether in the adult or child, they are peculiarly proper, especially if the fever may have habitually returned for several days, or there is reason to suspsct some additional source of irritation about the stomach or biliary organs, in such instances they cannot be too promptly administered. But not so in stupor or feeble old age, nor in yellow fever, are they to be administered.

"What are the effects of emetics, that they are so generally serviceable in fever?

"In the first place they empty the stomach of its contents; not only of indigestible food, but oftentimes of an inordinate quantity of mucus and other materials which may be accumulated in that organ. They promote the secretions of the stomach, and the adjacent viscera, particularly the liver. They increase the serous discharge of the intestines, and by the relaxing and antipasmodic effects on the whole system, they restore the perspiration, and unlock most of the secretions and excretions of the body. The emetic I prefer in such cases, and with the view to these general febrifuge effects, is composed of ipecac. and antim. as follows: Ipecac. x. gr. to xv. gr., combined with tart. antimony, ij. gr., directing the patient to take little or no drink until it has operated; to be repeated, if necessary, in half an hour, or an hour, if it has begun to operate.

"For the removal of the second mode of attack, where the patient suffers much more pain, a great sense of soreness, or is affected by rigors or convulsions, such remedies are indicated as will most immediately diminish irritation, and particularly such remedies as are best calculated to allay spasm. Opiates, the warm bath, and tepid drinks must be had recourse to, and are among the best remedies we can employ for this purpose. Opiates are useful by diminishing sense and motion.

"To children I administer ten drops of laudanum every half hour, in conjunction with the tepid bath. In the adult thirty drops with ℥ss. of the sp. mind. may be repeated every half hour, until the nervous system be composed. If the stomach be much disturbed, warm mint water will be the best vehicle for the laudanum. A general warm bath, as soon as it can be procured, ought also in such cases to be employed. In the meantime, however, the limbs may be wrapped up in fomentations composed of vinegar and water, one-third or one-fourth vinegar, and two or three parts water, but not spirituous fomentations; as before, paying attention to the temperature at which they are applied, lest more mischief than good arise from the manner of their application. It is no less important too, in applying them as far as possible to prevent the clothes of the patient, and the bed and bedding from becoming wet with the application made use of, as the patient afterwards is rendered liable to a chill from the cold and moisture surrounding him. For this purpose then, be careful to introduce one fold of the blanket between the legs of the patient and the bed he lies on. Let his limbs then be surrounded by the wet flannels with which the fomentation is applied, and another fold of the blanket that is beneath him turned, so as also to protect the clothing that is above him from becoming wet and uncomfortable. Another ready and common mode of applying steam or vapour to the body, and one to which the elder Dr. Bard was attached, is by means of bricks heated to a proper temperature. These should be surrounded first by a flannel cloth wet with a mixture of vinegar and water, and in order to prevent the bed from being made wet and uncomfortable, this should be again surrounded with another piece of dry flannel. Two of these bricks thus prepared and laid to the extremities, or at the sides of the patient, will be found useful both in restoring warmth, and inducing that greatest febrifuge of all, perspiration. And we may observe, that the temperature of the body is much more reduced by perspiration than augmented by the heat that is thus applied in conjuction with moisture or steam. So, in like manner, the effects of the warm bath in producing a large discharge from the surface, more than counteracts all the heat that has been applied in the bath. But as I have before remarked, you cannot, as a general rule, be too particular in your directions relative to the temperature of all the internal or external applications that may be necessary in the treatment of fever at this stage.

"But again: in old men much affected by cold and rigor, their caloric carried off, the patients feeble and not in condition readily to restore the lost heat, and the heat perhaps abstracted by long previous exposure to cold; in that case stimuli may be administered, and are indicated. But even then they should be directed with some caution. *Respice finem* should still be our motto. Cordials and aromatics are improper in most cases of the invasion of fever; but in those which I have just mentioned, in which the system is feeble, the action in the extreme vessels can only be restored by stimuli, administered both internally and externally. Warm and stimulating drinks, such as gin or brandy toddy, spiced wine, or wine whey, should now be freely administered, until the temperature of the patient be restored. With the same view, other stimulants may be prescribed; as a tea-spoonful of compound spirits of lavender, frequently repeated, or twenty or thirty drops of the vinous spirit of ammonia, or of the aq. ammoniæ.

"External applications should also be directed, as a hot bath, made still more exciting by the addition of rum, or the aq. ammon. occasionally introduced while the patient is immersed in the water. Stimulant spirituous fomentations—stimulant cataplasms, prepared by dipping a slice of toasted bread in hot vinegar, and covering the same with mustard, may be applied to the soles of the feet, or other sensible parts of the body. The room too should be rendered warm, the patient covered with rather more than the ordinary quantity of clothing, with the view to accumulate heat about his person. But remember, all these stimuli are again gradually to be withdrawn, in proportion as the excitement of the system becomes restored, or may be increased; otherwise these very means of restoring the heat and circulation will be the means of exciting a high and dangerous degree of febrile action.

"But when the third mode of the invasion of fever comes on with delirium, stupor, or mania, a method of treatment is called for totally different from that used in the ordinary attack, or in that accompanied with great coldness, severe pain, rigors, or convulsions. In this third mode of invasion, no cold stage is perceptible; it at once comes on with stupor, delirium, or the ravings of mania. In that case, if the pulse be full and slow, or hard and frequent, and the habit of body plethoric, as is frequently the case even in advanced life, we must have recourse to depletion. Not opium in this case; not, however, for the reason that perhaps some of you may give, because it proves a direct stimulant to the system; no, but because it retards the action in the smaller vessels, and thereby crowds the larger. Do you want proof of this? You have occular demonstration of it in the experiments made by Dr. Monro upon frogs. You also have it in the experiments made by Dr. Bard upon himself—see his thesis. But if it be not the property of a sedative to diminish sense and motion, then opium may be called a stimulant; and if it be the property of the stimulant to excite sense and motion, then opium is assuredly sedative.

"But to return. Evacuations by the lancet, and perhaps by cupping, or by dividing the temporal artery, if the symptoms be urgent—by cathartics, blisters, and sudorifics, (such as cream of tartar, sp. mind., &c.) to diminish the quantity of circulating fluids, which especially oppress or iritate the brain, must be employed. The vital functions being thus attacked, the most active measures become necessary.

"In the 188th No. of the Medical and Physical Journal, this practice is recommended as a new practice! and announced as a great discovery; and lately the same practice of blood-letting has been recommended in the treatment of typhus by Walsh of Edinburgh, and by Dr. Armstrong in his late work on the same subject, as a novel treatment. Venesection in intermittents, and in typhus, is a doctrine which has been well understood and practised in this country for many years, and taught in the University of Pennsylvania by Dr. Rush, and in this college. I also taught it in the very first lessons I ever delivered on the treatment of fever. All light does not proceed from the east. I had almost said, that Dr. Rush alone has done more towards introducing an efficient practice in the treatment of diseases, than all his cotemporaries in Europe or any other parts of the world collectively have done. But caution is no less necessary in discriminating between those cases where such active treatment is called for, and those where it is inadmissible; we otherwise may extinguish life instantaneously, for fever in this invasion is sometimes immediately fatal. But again; do not suffer the life of your patient to be sacrificed in this apoplectic form of fever for the want of the lancet, because forsooth it is symptomatic of the invasion of an intermittent, in which blood-letting in general is not advisable or necessary. Venesection, in the cold stage of an intermittent, has been lately recommended in the Edinburgh Journal, as if a new practice. It has been long since well understood in the United States. Be cautious, too, not to commit yourselves by denominating this form of fever apoplexy, for your patient in a few hours may be relieved, and the want of discrimination in you be censured for not foreseeing this result.

"Purges, in like manner, are generally improper in the first or cold stage of fever. So also, are such drinks as cream of tartar and tamarinds, &c., but they are not improper in the present state of stupor or phrenitis; they are now indicated. And recollect, too, that cathartics are not only indicated for the purpose of diminishing the fluids of the system, but also to transfer excitement from the brain, and thereby also to lessen the quantity of fluids there determined by such irritation. With this view, saline cathartics, or stimulant cathartics, composed of jalap and calomel, are to be preferred. I cannot, however, too severely reprobate the use of the small doses of calomel, usually and promiscuously prescribed under the appellation of fever powders; they are indeed fever powders, for they most effectually continue fever. Calomel, as a cathartic, in the beginning of fevers, is among the best that can be employed, and occasionally too, may be administered in the progress of the fever. It excites a degree of nausea, sometimes vomiting; unloads the biliary organs, dislodges scybala, invites a large secretion into the intestines; and afterwards, in unison with antimony, has a sensible effect upon the surface, as well as the excretory organs in general. Such purgatives as excite the whole system, as does colocynth, gamboge, &c., are to be avoided. This is where cathartics are indicated to empty the intestines, and thereby to prevent the absorption of the contents of the belly, which, as a means of repletion, would aggravate the disease, while by their qality they would add to the malignancy of such fever. Castor oil is frequently prescribed as a domestic purge in the beginning of fevers; as a means of emptying the intestinal tube it is effectual, but not so in its operation upon the liver, or the excretions of the general system. Enemata are also indicated, as more immediate in their operation than cathartic medicines.

"In the treatment of fever it frequently becomes necessary to combine all your forces, and as nearly as possible at the same time, and not in the successive and inefficient manner that we see remedies often directed. Such procedure is similar to that of a commander who suffers his army to be cut off, regiment by regiment; whereas, by one general engagement he might have been sure of victory, and that with a small loss of his men. Thus in fevers the powers of life are gradually destroyed by the continuance of the disease, and the many repeated, feeble and insufficient attempts in succession to bring about a cure—whereas, by summoning all your resources at the same time, you put the enemy to flight, and prevent that loss of strength that otherwise would be the result. We should not wait in such cases for the slow operation of cathartics, and which are rendered still more slow by the influence of the fever upon the system, but immediately administer the domestic injection, by which you will remove a great additional source of irritation from the bowels. Take oil, molasses, or honey; common salt, aa ℥ss.; water ℔.i. M.; or equal parts of milk and water; or soft soap, ℥ii., water ℔j.; or which is still more active, castor oil, ℥s., glauber salts, ℥s., aq. pluvial. ℔i. M.

"Blisters are another means of diverting the excitement from the brain to the surface of the body, not by the mere discharge of fluids they occasion, but by the excitement they produce upon the surface, or to the part to which they are applied. Such, too, is the opinion of Dr. Jackson, that they produce their good effects by the local affection they create.* Armstrong, too, highly approves of blisters, as among the means of breaking up febrile action. But even the derivation of the fluids in these cases is useful, by abstracting them from the brain to which they tend, and the sooner blisters are applied the better. They should be applied behind the ears, between the shoulders, to the wrists, to the ankles, or to the præcordia; that is, to the most sensible parts of the body, for the very purpose of creating new and powerful excitement.

Sudorifics also constitute an important class of remedies in this stage and state of fever—that is, such sudorifics as at the same time that they relax the surface of the body, also diminish excitement in general. The sp. mindereri—aq. acetat amm., may be advantageously combined with a portion of tart. antimony. Antimony, in some of its forms, especially tartarized antimony, the tartrite of antimony and potass. gr.

* Jackson on Fevers, 224.

ij. in eight doses, with cream of tartar, ʒij., or in sweetened water alone. Vin. antimoniale, xxx. gtt. to ʒi., the pulvis antimonialis, that is, the antim. calc. phosph. gr. iv. to gr. vi. every two hours, in syrup. The real James' powder, from gr. x. to gr. xv. or ℈i. every two or three hours, or combined with calomel, from gr. iij. to gr. vi. of each combined, repeated every three hours. These diaphoretic or sudorific medicines should also be aided in their operation by suitable drinks. When the patient is in the use of antimonial medicines, his drink should be some of the following:—toast water, catmint tea, (nepeta cataria,) balm tea, (melissa officinalis,) common tea, (thea viridis,) bran tea, rice or barley water. But when the patient is not in the use of antimonial medicines, he may make use of other drinks, which he will find both more grateful and useful during the heat and thirst of fever, as lemonade, apple water, tamarind water, currant jelly and water, molasses and water with the addition of a small quantity of vinegar; this is commonly called switchell by our eastern brethren, and a most excellent drink it is too in fevers, unless the bowels may be too freely opened. It is frequently directed by me in the hospital. Vinegar whey, too, is another valuable drink in this excited state of the system; but these acid drinks, taken during the use of antimonial medicines, are frequently attended with pain in the bowels and in some instances they render those medicines dangerously active. They should give place, therefore, to some of those before enumerated."

Presuming that the western student of medicine, and the western practitioner of good intellect and some year's experience, have carefully read and studied the foregoing "Lecture," we ask the former what he has learnt from it, of a decided, pointed, and definite character? and the latter, what he has met with in it, in accordance with the practice he has found it necessary himself to pursue? And we feel no apprehension, that the reply from either will materially conflict with the sentiments respecting the production, which we have ourselves so openly expressed in the preceding pages. On the contrary we are persuaded it will harmonize with them. We venture to say, that any intelligent, educated, and observing physician, twenty-five years of age, and of three years standing as a practitioner, in the Mississippi Valley, could instruct Professor Hosack, were he now living, how to *treat our prevailing fevers*, of every description, better—vastly better, than he instructed his class respecting them, by delivering his "Lectures" to them; or than his Reverend Editor will instruct the American faculty, by the publication of those "Lectures." Yet has Professor Chapman announced to the world that he "considers the *practical part* (of the "Lectures") *sound;* and that it corresponds very much with his own views." We do not pronounce the practice thus concurrently recommended by Professors Hosack and Chapman unsound, as relates to the complaints of the Eastern States. Far otherwise. On that point it would be unbecoming in us to deliver our opinion. But, as respects the complaints of the Western States, we do so pronounce it, because we *know* it to be, unsound. To teach it therefore to pupils, who intend to pursue their profession in the West and South, is to mislead and injure them. Worse still; it is to put the lives of their patients in danger.

There are also contained in the Lectures before us certain pathological doctrines, which we deem not only groundless in themselves, but, as far as their influence may extend, pernicious in their consequences. Prominent in this respect are those which relate to *contagion* and *humoralism*—especially that form of humoralism, of which a supposed putrescent condition of the human fluids during life, constitutes an element. These doctrines, connected with a few other points of interest, will probably be made the basis, at a future period, of another critique on the work we are considering. Meantime we say distinctly now, that we regard the unfounded notions respecting contagion and putresency, contended for by the late Professor Hosack and a *few* other physicians, to be, in their practical effects, among the most mischievous that have been broached by medical writers. And we further say, that there is no physician, whether he be a Professor or not,whose sphere of practice has been exclusively east of the Alleghany mountains, who is competent either to deliver lectures, or to write a book, containing correct instructions how to treat the complaints of the West. Lectures and books, containing such instructions, can be prepared only by physicians who have a *practical* acquaintance with such complaints. If there be *positive maxims* in medical science, this is one of them. And it has been long a matter of regret and surprise to us, that due action has not been taken on it by western physicians.

